# Sample size determination for estimating antibody seroconversion rate under stable malaria transmission intensity

**DOI:** 10.1186/s12936-015-0661-z

**Published:** 2015-04-03

**Authors:** Nuno Sepúlveda, Chris Drakeley

**Affiliations:** London School of Hygiene and Tropical Medicine, Keppel Street, WC1E 7HT London, UK; Center of Statistics and Applications of University of Lisbon, Faculdade de Ciências da Universidade de Lisboa, Bloco C6 - Piso 4, 1749-1016 Lisboa, Portugal

**Keywords:** Seroprevalence, Seroconversion rate, Bias, Precision, Sample size

## Abstract

**Background:**

In the last decade, several epidemiological studies have demonstrated the potential of using seroprevalence (SP) and seroconversion rate (SCR) as informative indicators of malaria burden in low transmission settings or in populations on the cusp of elimination. However, most of studies are designed to control ensuing statistical inference over parasite rates and not on these alternative malaria burden measures. SP is in essence a proportion and, thus, many methods exist for the respective sample size determination. In contrast, designing a study where SCR is the primary endpoint, is not an easy task because precision and statistical power are affected by the age distribution of a given population.

**Methods:**

Two sample size calculators for SCR estimation are proposed. The first one consists of transforming the confidence interval for SP into the corresponding one for SCR given a known seroreversion rate (SRR). The second calculator extends the previous one to the most common situation where SRR is unknown. In this situation, data simulation was used together with linear regression in order to study the expected relationship between sample size and precision.

**Results:**

The performance of the first sample size calculator was studied in terms of the coverage of the confidence intervals for SCR. The results pointed out to eventual problems of under or over coverage for sample sizes ≤250 in very low and high malaria transmission settings (SCR ≤ 0.0036 and SCR ≥ 0.29, respectively). The correct coverage was obtained for the remaining transmission intensities with sample sizes ≥ 50. Sample size determination was then carried out for cross-sectional surveys using realistic SCRs from past sero-epidemiological studies and typical age distributions from African and non-African populations. For SCR < 0.058, African studies require a larger sample size than their non-African counterparts in order to obtain the same precision. The opposite happens for the remaining transmission intensities. With respect to the second sample size calculator, simulation unravelled the likelihood of not having enough information to estimate SRR in low transmission settings (SCR ≤ 0.0108). In that case, the respective estimates tend to underestimate the true SCR. This problem is minimized by sample sizes of no less than 500 individuals. The sample sizes determined by this second method highlighted the prior expectation that, when SRR is not known, sample sizes are increased in relation to the situation of a known SRR. In contrast to the first sample size calculation, African studies would now require lesser individuals than their counterparts conducted elsewhere, irrespective of the transmission intensity.

**Conclusions:**

Although the proposed sample size calculators can be instrumental to design future cross-sectional surveys, the choice of a particular sample size must be seen as a much broader exercise that involves weighting statistical precision with ethical issues, available human and economic resources, and possible time constraints. Moreover, if the sample size determination is carried out on varying transmission intensities, as done here, the respective sample sizes can also be used in studies comparing sites with different malaria transmission intensities. In conclusion, the proposed sample size calculators are a step towards the design of better sero-epidemiological studies. Their basic ideas show promise to be applied to the planning of alternative sampling schemes that may target or oversample specific age groups.

**Electronic supplementary material:**

The online version of this article (doi:10.1186/s12936-015-0661-z) contains supplementary material, which is available to authorized users.

## Background

Parasite prevalence (PR) and entomological inoculation rate (EIR) are the two most common disease risk indicators used in malaria epidemiology. PR is defined as the percentage of people who are currently infected with malaria parasites, and reflects the direct interplay between transmission intensity, age, and disease burden. EIR is in turn the frequency at which people are bitten by infectious mosquitoes over a period of time (typically a year), and provides information on the vector biology and its interaction with the human host. These measures, although useful in high and moderate transmission settings, show limitations in areas of lower transmission or in populations on the cusp of disease elimination. This is primarily due to the low number of infected individuals (humans or mosquitoes) in the population at the time of sampling. Accurate metrics are particularly important in assessing the effects of malaria interventions at these low transmission levels. Therefore, in recent years, alternative risk indicators based on anti-malarial antibody seroprevalence (SP) and seroconversion rate (SCR) have been evaluated [1-4].

The rationale of using antibody data stems from the observation that specific antibodies against parasite antigens persist in time and at reasonably stable concentrations, even when disease transmission is seasonal. Experimentally, the quantification of antibodies in sera is relatively easy to perform using simple laboratory techniques, such as ELISA assays. The resulting antibody measurements are usually optical densities or the respective titre values upon which one classifies each individual as seronegative or seropositive using appropriate cut-off points. These seropositivity thresholds are typically determined by two distinct approaches. The first one uses antibody data of known seronegative individuals in which the parameters of the underlying distribution are estimated, as illustrated by Arnold *et al.* [5]. In contrast, the second approach is based on fitting a Gaussian mixture model to current antibody data directly under the assumption that there are two latent subpopulations referring to seronegative and seropositive individuals, respectively [6]. In both approaches, the cut-off point for seropositivity is determined by the average plus 3 times the standard deviation of the seronegative population. Seroprevalence (SP) is then the percentage of seropositive individuals in the sample and embodies information over currently infected and recently exposed individuals. As expected, SP estimates are typically higher than those for PR measured in the same sample [1,7]. Although overcoming some of the shortcomings of PR and EIR, SP does not reflect the dynamics of malaria transmission directly.

Seroconversion rate (SCR) extends SP analysis to the scenario where one is a step closer to capture the underlying disease dynamics of a given population. This serological parameter arises from the analysis of seroprevalence taken as function of age of the individuals using the so-called reverse catalytic models. The age of individuals is assumed to be a good surrogate of time in a stochastic process where individuals transit between seropositive and seronegative states upon malaria exposure and absence of re-infection. Theoretically, SCR is defined as the frequency by which seronegative individuals become seropositive upon malaria exposure. Conversely the frequency by which seropositive individuals return to a seronegative state is known as seroreversion rate (SRR). This last parameter is related to antibody decay in absence of disease exposure and reflects the effects of host factors on antibody dynamics.

Several studies have shown the utility of SCR as a malaria epidemiological tool with some demonstrating good agreement between this measure and EIR [1] and others detecting historical changes in transmission that otherwise would not have been possible with other measures of transmission [4,7-9]. Whilst the evidence for using serology as an adjunct epidemiological marker for malaria transmission is growing, there has been no formal examination of samples size considerations for SP and SCR as primary endpoints. In fact, most malaria epidemiological studies are planned with PR being as the primary endpoint [7] and, therefore, it is unclear whether SP and SCR might have enough statistical precision to lead to clear conclusions.

SP is in theory a proportion (or a percentage) and, as such, several methods exist for sample size determination in this situation [10]. In contrast, the precision of SCR estimates depends not only on the sample size, but also on the age distribution associated with a given population. Therefore, sample size determination is not as straightforward. A pragmatic approach is to use an empirical relationship between SCR and SP in order to determine the total sample size required for collecting a given number of seropositive individuals [8]. This approach is here improved by using the theoretical relationship between SP and SCR under a given age distribution and a fixed SRR. Sample size determination is then based on back-transforming the confidence interval for SP into the corresponding one for SCR. In the situation where SCR and SRR are both unknown, a second sample size calculator is developed by bringing simulation together with regression. The use of these two sample size calculators is instrumental to power future serological studies, notably, in the challenging research settings of populations on the cusp of elimination [11].

## Methods

### Reverse catalytic models for seropositivity data

In malaria epidemiology, the reverse catalytic models were first described to estimate incidence and recovery rates from longitudinal data [12]. More recently, they were recast to the analysis of malaria seroprevalence data [13]. Mathematically, these models can be described as a Markov chain where individuals transit between two serological states: 0 - seronegative and 1 - seropositive. The time between transitions is assumed to be exponentially distributed. This assumption implies that every time an individual move from one state to another, the stochastic process restarts probabilistically due to lack of memory of the Markov Chains. This is in close agreement with the general notion that malaria parasites can only confer partial immunity to the host.

This paper deals with the simplest reverse catalytic model where SCR and SRR are assumed to be fixed constants throughout time and for every individual. The use of this model has in practice three key implications. Firstly, a constant SCR implies that disease transmission remained unchanged throughout time in the population under study. Secondly, a constant SRR implies that the host factors affecting antibody decay were not altered by any genetic selection event, migration or admixture. Thirdly, all individuals have experienced the same disease transmission intensity and, thus, age can be used as a surrogate of the time of disease dynamics. Mathematically, the probability of individuals with age *t* being at each serological state is given by the transition probability matrix *P*(*t*) = [*p*_*i*|*j*_(*t*)], *i*, *j* = 0, 1, where *p*_*i*|*j*_(*t*) is the conditional probability of an individual with age *t* being in state *i* given he started the process in state *j* and *R* is the so-called rate matrix that, in turn, is defined as

1 $$ R=\left[\begin{array}{cc}\hfill -\lambda \hfill & \hfill \lambda \hfill \\ {}\hfill \rho \hfill & \hfill -\rho \hfill \end{array}\right], $$

where *λ* and *ρ* are the SCR and SRR, respectively. Assuming that all individuals are born seronegative (that is, seronegative at time *t =* 0; this is achieved in practice by only including individuals aged or older than 1 year to negate putative maternal effects on malaria antibodies), the probability of an individual aged *t* being seropositive is described by

2 $$ {p}_{1\Big|0}(t)=\frac{\lambda }{\lambda +\rho}\left(1-{e}^{-\left(\lambda +\rho \right)t}\right). $$

A special case of the above model may arise from populations where only a few seronegative individuals would result from seroreversion events. As a consequence, data might not enough information to estimate SRR (i.e., *ρ* ≈ 0). In this case, equation (2) can be rewritten as follows

3 $$ \log \left[- \log \left(1-{p}_{1\Big|0}(t)\right)\right]= \log \lambda + \log t. $$

This model has been applied to malaria data from low transmission populations [14], to serology data on human leishmaniasis [15], and to limiting dilution data [16]. Theoretically, equation (3) can be seen as the popular complementary log-log model from statistics that, in turn, can be formulated as a generalized linear model (GLM) under a binomial sampling scheme [17]. As such, the respective parameter estimation can be performed in most statistical softwares as long as one specifies 'log age' as the explanatory variable and the corresponding slope fixed at 1. Alternative sample size calculators for this model could be used in the same line of a GLM power analysis, as described elsewhere for logistic regression [18,19].

There are also other reverse catalytic models describing changes in disease transmission (see, for example, review of Corran *et al.* [1]). Although interesting, sample size determination on these alternative models will be studied elsewhere (Sepúlveda and Drakeley, in preparation). In malaria literature, one can also found an extension of the reverse catalytic modelling framework to the situation where seropositivity can be boosted by recurrent malaria exposure [20]. This model would appear to be more adequate to very high transmission settings and, thus, out of the scope of this paper.

### Model parameterization

To illustrate the sample size determination on realistic values of SCR and SRR, *Plasmodium falciparum* data sets from two independent studies in northeast Tanzania were used [3,21]. This region extends from the high malaria transmission areas in the coastal plains of Tanga to the low transmission settings in the high altitude mountains of Kilimanjaro, Usambara and Pare. Because of this natural variation in malaria endemicity, northeast Tanzania is an ideal region to understand how different malaria risk indicators are related to each other. Available data of altitude (in meters) against EIR [21] was re-analysed leading to the following linear regression model (Additional file 1: Figure A)

4 $$ { \log}_{10}\mathrm{E}\mathrm{I}\mathrm{R}=2.5204-0.0025\times \mathrm{altitude}. $$

In another epidemiological study, serological data from 21 villages of the same region was also available [3,13]. SCR associated with MSP1 antibodies was found to be highly correlated with altitude [1]. This data set suggested the following relationship between SCR and altitude (Additional file 1: Figure B)

5 $$ { \log}_{10}\mathrm{S}\mathrm{C}\mathrm{R}=-0.2908-0.0012\times \mathrm{altitude}, $$

where SRR estimate would appear to be constant across villages and fixed at 0.017. In turn, data from the same study suggested the following relationship between PR of children aged 0–4 years old (PR_04_) and altitude (Additional file 1: Figure C):

6 $$ \log \frac{{\mathrm{PR}}_{04}}{{1\hbox{-} \mathrm{P}\mathrm{R}}_{04}}=8.9992-1.5934\times { \log}_{10}\mathrm{altitude}. $$

**Figure 1:**
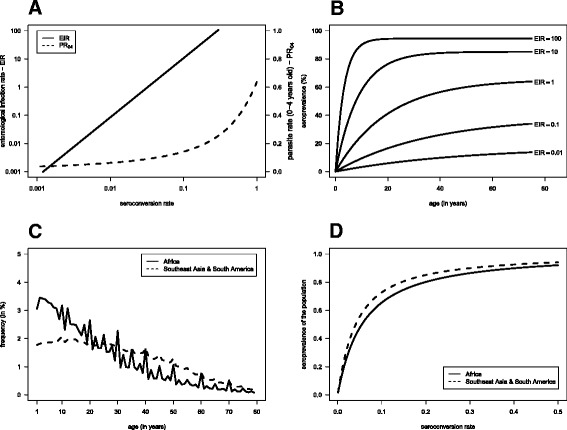
**Model parameterization under the assumption of constant malaria transmission intensity: A.** Expected relationship between SCR, EIR rate and PR in children aged from 0 to 4 years olds. **B.** Age-adjusted SP curves given the expected SCRs associated with EIRs shown in **A**. **C.** Age structure of African and non-African populations. **D.** Seroprevalence as function of SCR based on the age distributions shown in **C**.

Solving one of the above equations as function of altitude, the expected relationship between EIR, SCR, and PR_04_ can be obtained as shown in Figure 1A.

Sample size determination was conducted on the following transmission intensities as measured by EIR and PR_04_ (in brackets) units: 0.01 (0.050), 0.1 (0.073), 1 (0.119), 10 (0.231) and 100 (0.625). The corresponding SCRs are 0.0034, 0.0104, 0.0324, 0.0969 and 0.2900, respectively (Table 1). With respect to the above-mentioned large epidemiological study [1], a SCR between 0.0034 and 0.0104 describes low transmission intensities of high-altitude villages, such as Kilomeni (1556 m - SCR = 0.0047) or Mokala (1702 m - SCR = 0.0104). SCRs between 0.01 and 0.10 are, in turn, associated with villages in intermediate altitude, like Tewe (1049 m - SCR = 0.0308) or Ngulu (831 m - SCR = 0.0906). Finally, SCRs greater than 0.10 are related to lowland villages, such as Mgila (375 m - SCR = 0.128) or Mgome (196 m - SCR = 0.302), where malaria transmission is considered to be high. The expected age-adjusted SP curves are shown in Figure 1B.

**Table 1:** **Expected relationship between EIR, PR**_**04**_ **, SCR and SP in African (AFR), Southeast Asian and South American (SEA + SA) populations where seroreversion rate was fixed at 0.017**

**EIR**	**PR**_**04**_	**SCR**	**Seroprevalence**	
			**AFR**	**SEA + SA**
0.01	0.050	0.0036	0.057	0.073
0.10	0.073	0.0108	0.156	0.195
1.00	0.119	0.0324	0.365	0.437
10.0	0.231	0.0969	0.647	0.720
100.0	0.625	0.2900	0.860	0.896

### Model estimation

In terms of statistical analysis, age-adjusted seropositivity data can be summarized as a frequency vector {*n*_*ts*_} where *n*_*ts*_ is the frequency of individuals with age *t = 1,…,T* and serological state *s =* 0 or 1, *T* is the total number of distinct age values in the sample. If individuals were sampled independently of each other and the statistical inference is focused on age-adjusted seroprevalence only, the sampling distribution of the frequency vector {*n*_*ts*_} can be described by a binomial-product distribution, one binomial distribution per age value, that is,

7 $$ f\left(\left\{{n}_{ts}\right\}\Big|\lambda, \rho \right)={\displaystyle \prod_{t=1}^T\frac{\left({n}_{t0}+{n}_{t1}\right)!}{n_{t0}!{n}_{t1}!}}{\left[{p}_{1\Big|0}(t)\right]}^{n_{t1}}{\left[1-{p}_{1\Big|0}(t)\right]}^{n_{t0}}, $$

where *p*_1|0_(*t*) is given by equation (2). Parameter estimation can be performed via standard maximum likelihood methods, as described elsewhere [15]. Stata and R scripts for parameter estimation are available from the authors upon request.

### Sample size calculations

The first sample size calculator assumes that SRR is a known constant (say *ρ*_0_ = 0.017), thus, should not be estimated after sample collection. In that case, the expected relationship between SP of the population (hereafter denoted by *π*) and SCR can be computed as follows

8 $$ \pi ={\displaystyle \sum_{t=1}^{A_{\max }}{\alpha}_t\frac{\lambda }{\lambda +{\rho}_0}\left(1-{e}^{-\left(\lambda +{\rho}_0\right)t}\right),} $$

where *α*_*t*_ is the proportion of individuals aged *t* in the population and *A*_*max*_ is the maximum age considered relevant for the population, say *A*_*max*_ =80. As expected, the above relationship depends on the age distribution of the population (or of the study design used). Official statistics on age distributions were explored in order to understand how these vary across the world [22]. These data sets suggest that African countries have the same age distribution approximately (a decreasing frequency from newborns to elderly; Additional file 2). Thus, a typical age structure distribution for these populations was generated by pooling data from different countries together (Figure 1C). Although slight differences can be observed across countries, the age distributions from Southeast Asia and South America show roughly the same pattern but distinct from the one for African populations (Additional file 2). Therefore, a non-African age distribution prototype was constructed (Figure 1C). This age structure is much flatter than its African counterpart due to a higher frequency of adults.

These two general age distributions were then used to derive the expected SP as function of SCR according to equation (8) (see Figure 1D). Interestingly, the relationship between SP and SCR in African populations when SCR = 0 is similar to the one for non-African populations when *ρ* = 0.017. Therefore, the sample size determination would lead to similar results for these two distinct populations.

In the statistical literature, there are several methods for constructing a confidence interval for a proportion that can be used for sample size determination, as reviewed elsewhere [23]. The most popular method is the so-called Wald Score that, although its simplicity of calculation, may lead to poor coverage and problems of overshoot and degeneracy [10]. An alternative method is to introduce an continuity correction in the Wald Score that, when applied to SP estimation, leads to the following confidence interval at 95%

9 $$ {\widehat{\pi}}_l=\widehat{\pi}-1.96\sqrt{\frac{\widehat{\pi}\left(1-\widehat{\pi}\right)}{n}}-\frac{1}{2n} $$

and

10 $$ {\widehat{\pi}}_l=\widehat{\pi}+1.96\sqrt{\frac{\widehat{\pi}\left(1-\widehat{\pi}\right)}{n}}+\frac{1}{2n}, $$

where $\widehat{\pi}$ is an estimate of the true SP, *n* is the sample size and 1.96 is the 97.5%-quantile of a standard Gaussian distribution. For a given SCR, one can compute the expected *π* using equation (8) and replace it in the above equations in order to obtain the corresponding confidence bounds ${\widehat{\pi}}_l$ and ${\widehat{\pi}}_u$ for a given sample size *n*. These confidence bounds can then be back-transformed into the corresponding ones for SCR using equation (8) again. To perform the back-transformation, one needs to solve the following equations as function of *λ*_*l*_ and *λ*_*u*_ (the corresponding lower and upper bounds of SCR)

11 $$ {\widehat{\pi}}_l={\displaystyle \sum_{t=1}^{A_{\max }}{\alpha}_t\frac{\lambda }{\lambda +{\rho}_0}\left(1-{e}^{-\left(\lambda +{\rho}_0\right)t}\right),} $$

and

12 $$ {\widehat{\pi}}_u={\displaystyle \sum_{t=1}^{A_{\max }}{\alpha}_t\frac{\lambda }{\lambda +{\rho}_0}\left(1-{e}^{-\left(\lambda +{\rho}_0\right)t}\right).} $$

Unfortunately these equations can be solved analytically but a binary searching algorithm, although slow, is able to obtain an approximate solution using an appropriate searching interval.

In theory, one defines the coverage of a confidence interval as the number of times that confidence interval contains the true value of the parameter upon repeated sampling. Under this definition, a confidence interval at 95% should lead to a coverage of 95%. However, the expected coverage is not always achieved due to the use of (Gaussian) approximations for the random variables underpinning the construction of a given confidence interval. This putative incorrect coverage affects sample size determination by either undersampling in situations of undercoverage or oversampling in situations of overcoverage, as reported for proportion estimation when data stems from populations with proportions less than 0.1 or higher than 0.9 [23,24]. Therefore, the back-transformation method was tested against these putative coverage problems.

The expected coverage of the confidence interval for SCR was assessed via simulation. For every pairwise combination of SCR and *n*, the following two-step algorithm was employed for the generation of a given data set: i) generate the age of each individual in the sample, and (ii) generate the corresponding serological state as a Bernoulli trial with seropositivity probability given by equation (2). The back-transformation of the confidence interval for SP was applied to each data set. Coverage was finally calculated by counting how many times the confidence intervals included the SCR that generated the data.

The performance of this method was also assessed in terms of the midpoint of the corresponding confidence interval for SCR. In this scenario, a confidence interval was defined as central if the true SCR was located in the middle of the corresponding interval. A practical implication of using central confidence intervals is that they have the shortest length among all intervals one can construct with a given confidence level if a Gaussian distribution is a good approximation for the sampling distribution of SCR estimates. In that case, the use of central confidence intervals for sample size determination implies working with the best precision possible and, thus, the subsequent sample sizes are the minimum ones for a given confidence level. In opposition, if the constructed confidence intervals are not central, they might not be the ones providing the highest precision (i.e., with shortest length). To assess whether a given confidence interval is or not central, one is required to know the sampling distribution of SCR estimates upon repeated sampling. Unfortunately that distribution is not known in general.

Sample size determination was then conducted by given length of the 95% confidence interval for SCR. With this goal in mind, the relative length of that confidence interval was fixed at a given constant (*e.g.*, 1, 0.75, 0.5, and 0.25). The above back-transformation method was used together with an additional binary search method aiming to find the required sample size. The search algorithm was implemented in R software and the corresponding code is available from the authors upon request.

When there is little information on SRR to help planning a study, there is no clear analytical method to calculate the required sample size. Instead, data simulation would appear to be the best approach for the problem. Specifically, data simulation was used to study the expected length of the confidence intervals for SCR given a set of sample sizes (*e.g.*, *n* = 250, 500, 1,000, 2,500, 5,000 and 10,000). The generation of each data set followed the same algorithm as described for the performance of the first sample size calculator. For each generated data set, the estimates of SCR and SRR were obtained via maximum likelihood methods. To obtain the precision of SCR estimate associated with a given sample size, the 2.5% and 97.5% quantiles were calculated for the set of SCR estimates generated from data of a given transmission intensity. The absolute precision was defined as the absolute difference between these two quantiles whereas the relative precision is the absolute precision divided by the SCR that generated the data.

It is worth noting that the absolute precision (pr) of SP estimates associated with the first sample size calculator can be rewritten as a function of *1/n* given a pair of SCR and SRR, that is,

13 $$ {\mathrm{pr}}_{n\Big|{\rho}_0}\left(\widehat{\pi}\right)=3.92\sqrt{\frac{\widehat{\pi}\left(1-\widehat{\pi}\right)}{n}}+\frac{1}{n}, $$

where the above equation results from the absolute difference between equations (9) and (10). Since this sample size calculator is based on a back-transformation relating SP to SCR, the precision of SCR estimates can also be expressed by a function of *1/n* (say function *g*). This function is highly non linear and not analytically derivable but in theory can be approximated by the following MacLaurin expansion from Mathematical Calculus:

14 $$ {\mathrm{pr}}_{n\Big|{\rho}_0}=g(0)+\frac{g\hbox{'}(0)}{1!}\times \frac{1}{n}+\frac{g\hbox{'}\hbox{'}(0)}{2!}\times \frac{1}{n^2}+\frac{g\hbox{'}\hbox{'}\hbox{'}(0)}{3!}\times \frac{1}{n^3}+\cdots $$

where *g* ′ (0), *g* ′′ (0) and *g* ′′′ (0), are unknown but fixed constants associated with the function *g*, its first, second and third derivative evaluated at zero, respectively. Therefore, the precision of SCR estimates ($\widehat{\lambda}$) can be determined by a regression linear model as function of *1/n*, that is,

15 $$ p{r}_{n\Big|{\rho}_0}\left(\widehat{\lambda}\right)={\beta}_0+\frac{\beta_1}{n}+\frac{\beta_2}{n^2}+\frac{\beta_3}{n^3}, $$

where *β*_0_, *β*_1_, *β*_2_ and *β*_3_ are coefficients to be estimated from the set of SCR estimates obtained from the simulated data. This rationale was assumed to be applicable directly to the second sample size calculator where SRR is unknown. The above model was then estimated to the simulated precision data via maximum likelihood method. The resulting adjusted correlation coefficient between simulated and predicted data was found to be >0.99, thus, suggesting that the above model is indeed a good approximation of the relationship between the sample size and the expected precision of SCR estimates. The last step was to find the sample size associated with a given precision. This was done numerically by using a binary search algorithm.

## Results

### Performance of the back-transformation method

The performance of the back-transformation method was first assessed in terms of the expected coverage of the 95% confidence intervals for SCR (Table 2). In most cases, the confidence intervals showed slight overcoverage (≤1%) with a few exceptions. In very low transmission settings (SCR = 0.0036), the confidence intervals show undercoverage for sample sizes ≤250 in Africa and ≤500 elsewhere, respectively. The most severe case of incorrect coverage is for samples of 50 individuals from African populations where a strong overcoverage (0.998) is observed. Interestingly, in a non-African context, the confidence intervals show instead undercoverage (0.909) for the sample size and transmission intensity. These opposing results might reflect marked differences in the underlying age structures, notably in terms of the proportion of children in one population and the other (see Figure 1C). In high transmission intensities (SCR = 0.29), the confidence intervals also show undercoverage for samples of 100 individuals or less in African settings. In practice, the problem of under or overcoverage most likely results in confidence intervals with higher or lower length than they should in relation to a situation where the correct coverage is obtained for the constructed intervals. This has an impact on sample size determination in the sense that controlling the length of the confidence intervals showing these problems might lead to smaller or greater samples sizes than required in reality.

**Table 2:** **Coverage of confidence intervals based on back-transformation algorithm assuming SRR = 0.017**

**Population**	**Sample size**	**Seroconversion rate**				
		**0.0036**	**0.0108**	**0.0324**	**0.0969**	**0.2900**
Africa	50	0.998	0.958	0.960	0.959	0.922
	100	0.930	0.948	0.950	0.951	0.937
	250	0.939	0.956	0.959	0.952	0.951
	500	0.958	0.952	0.956	0.955	0.957
	1,000	0.954	0.958	0.955	0.957	0.951
Elsewhere	50	0.909	0.942	0.952	0.954	0.970
	100	0.935	0.953	0.958	0.954	0.951
	250	0.948	0.955	0.958	0.961	0.954
	500	0.946	0.958	0.954	0.955	0.955
	1,000	0.954	0.953	0.955	0.952	0.952

Confidence intervals for SCR estimates were then evaluated in terms of their midpoints. The results suggest that these midpoints and the true SCR tend to be closer to each other with the increase of the sample size (Additional file 3: Figure A). Mathematically speaking, this results from approximating the back-transformation by means of a linear relationship between SP and SCR. The precise sample size where that begins to happen increases with the underlying transmission intensity. More specifically, sample sizes of about 400 and 2,250 individuals tend to provide central confidence intervals when SCR=0.0036 and 0.29, respectively. For moderate sample sizes, say *n* < 500, the back-transformation method implies non-central confidence intervals for intermediate values of SCR. Since the exact distribution of SCR estimates is not known in general, it is unclear whether these non-central confidence intervals are the ones providing the highest precision.

### Sample size calculations for known SRR

Sample size determination was then conducted under the assumption of a known SRR (SRR = 0.017; Table 3). For the same relative precision, the sample sizes vary with transmission intensities. In particular, sample sizes increase from very low to intermediate transmission intensities and then they declined after reaching a sufficiently high transmission intensity (*i.e.,* when the SP curve becomes flat). With the increase of precision, the difference between sample sizes from different transmission intensities increases dramatically. On one extreme, for a relative length of 1, sample sizes vary from 73 (SCR = 0.0324) to 315 (SCR = 0.0036) and from 67 to 248 in African and non-African settings, respectively. On the other extreme, sample sizes range from 976 to 4968 (Africa) and from 890 to 3558 (elsewhere) for a relative length of 0.25.

**Table 3:** **Exact sample sizes and corresponding ranges for absolute SCR, EIR and SP by controlling the relative length of 95% confidence interval for SCR under the assumption of SRR = 0.017**

**Population**	**Relative length**	**SCR**	**Sample size**	**SCR**	**EIR**	**SP**
Africa	1.00	0.0036	315	0.0019-0.0054	0.00-0.03	0.030-0.085
		0.0108	128	0.0058-0.0166	0.03-0.26	0.090-0.223
		0.0324	73	0.0189-0.0513	0.34-2.75	0.248-0.483
		0.0969	79	0.0627-0.1594	4.18-29.17	0.536-0.759
		0.2900	151	0.1994-0.4885	46.5-300.58	0.801-0.919
	0.75	0.0036	549	0.0023-0.0050	0.00-0.02	0.037-0.078
		0.0108	220	0.0070-0.0151	0.04-0.21	0.106-0.207
		0.0324	123	0.0218-0.0461	0.46-2.20	0.276-0.455
		0.0969	127	0.0689-0.1415	5.08-22.76	0.560-0.735
		0.2900	233	0.2137-0.4307	53.7-231.22	0.813-0.907
	0.50	0.0036	1163	0.0027-0.0045	0.01-0.02	0.044-0.071
		0.0108	479	0.0082-0.0136	0.06-0.17	0.123-0.190
		0.0324	264	0.0250-0.0412	0.61-1.74	0.305-0.425
		0.0969	262	0.0765-0.1249	6.31-17.54	0.588-0.707
		0.2900	461	0.2326-0.3776	64.05-175.78	0.827-0.893
	0.25	0.0036	4,968	0.0032-0.0041	0.01-0.01	0.051-0.064
		0.0108	1,878	0.0095-0.0122	0.08-0.14	0.140-0.173
		0.0324	1,009	0.0285-0.0366	0.81-1.36	0.335-0.396
		0.0969	976	0.0858-0.1100	8.02-13.46	0.617-0.678
		0.2900	1,670	0.2576-0.3301	79.28-132.89	0.843-0.877
Elsewhere	1.00	0.0036	248	0.0019-0.0055	0.00-0.03	0.039-0.107
		0.0108	105	0.0059-0.0166	0.03-0.26	0.115-0.276
		0.0324	67	0.0195-0.0516	0.37-2.79	0.311-0.563
		0.0969	90	0.0645-0.1612	4.43-29.85	0.622-0.819
		0.2900	209	0.2019-0.4915	47.71-304.46	0.853-0.940
	0.75	0.0036	439	0.0023-0.0050	0.00-0.02	0.047-0.098
		0.0108	179	0.0070-0.0151	0.04-0.21	0.134-0.256
		0.0324	111	0.0221-0.0463	0.48-2.22	0.340-0.534
		0.0969	142	0.0700-0.1426	5.26-23.12	0.643-0.798
		0.2900	320	0.2154-0.4325	54.58-233.33	0.861-0.931
	0.50	0.0036	953	0.0027-0.0045	0.01-0.02	0.056-0.090
		0.0108	388	0.0082-0.0136	0.06-0.17	0.154-0.236
		0.0324	234	0.0251-0.0413	0.62-1.75	0.371-0.503
		0.0969	288	0.0771-0.1254	6.41-17.7	0.667-0.774
		0.2900	629	0.2335-0.3784	64.59-176.58	0.872-0.921
	0.25	0.0036	3,558	0.0031-0.0040	0.01-0.01	0.064-0.082
		0.0108	1,507	0.0095-0.0122	0.08-0.14	0.175-0.216
		0.0324	890	0.0286-0.0367	0.81-1.36	0.404-0.470
		0.0969	1,059	0.0859-0.1101	8.04-13.49	0.693-0.748
		0.2900	2,263	0.2579-0.3304	79.44-133.1	0.884-0.909

Similar sample sizes were found for African and non-African populations experiencing SCR = 0.0324 and 0.0964 (intermediate transmission) irrespective of the relative precision used. When SCR = 0.0964, the sample sizes for African populations are 79, 127 and 262 and 976 individuals to ensure a relative precision of 1, 0.75, 0.5, and 0.25, respectively, whereas the corresponding ones for non-African settings are 90, 142, 288 and 1,059. However, African studies require larger sample sizes than their non-African counterparts for SCR = 0.0036 and 0.0108 and the other way around for SCR = 0.29. For the same transmission intensity, the requirement of a smaller or larger sample size in African studies in the relation to others conducted elsewhere reflects the steepness of the SCR-SP curve. In other words, the use of the back-transformation implies that, when specifying a given confidence interval for SP, the confidence interval for SCR is going to be narrower or wider depending on the steepness of the SP curve. Mathematically, the steepness of that curve is given by the respective derivative. That derivative was found to be smaller in African than in non-African populations for SCR < 0.058 and the other way around for SCR > 0.058 (Additional file 3: Figure B). Available PR data for *P. falciparum* suggests that non-African populations are most likely to be at lower endemicity [25]. Note that, for SCRs in the vicinity of 0.058 where the two derivative functions cross each other, it is expected to obtain similar sample sizes for both populations, a result compatible with the sample sizes provided for intermediate transmission intensities. Finally, the relationship between SCR and SP was here found to be similar between Africa and non-African populations when SRR = 0 and 0.017, respectively (Figure 1D). Therefore, the comparison between sample sizes for African and non-African studies can also be used to ascertain the bias in sample size estimates when assuming SRR = 0 in an African setting.

The calculated sample sizes can also be used to help designing studies including different populations (or sites). Firstly, there is no theoretical impediment to use distinct sample sizes for populations known to differ in malaria endemicity. For example, a sample size of approximately 125 individuals will provide a relative precision of 1 for African sites experiencing a SCR of 0.0108. The same sample size leads to a relative precision of 0.75 for African populations with SCR = 0.0324 or 0.0969. Secondly, the expected confidence intervals for SCR can also provide clear insights on the underlying statistical power to compare sites with different transmission intensities. In particular, the sample sizes associated with a relative precision of 1 are enough to distinguish sites differing at least one order of magnitude in EIR with 95% confidence (or with 5% significance level in hypothesis testing terminology). However, this distinction cannot be done if these sample sizes were used and a 99% confidence level was alternatively specified to study between any two sites differing exactly one order of magnitude (Additional file 4). Thirdly, the expected confidence intervals for SCR are alternatively instrumental to know which transmission intensity range cannot be discriminated by the data. For example, a sample size of 79 individuals associated with a relative length of 1 and SCR = 0.0969 cannot distinguish African populations with EIR ranging from 4.18 to 29.17.

### Sample size calculations for unknown SRR

Sample size calculations were then performed for the most common situation of unknown SRR. For low transmission settings (SCR ≤ 0.0108) and reasonably low sample sizes, there is a non-negligible probability of generating data sets leading to null SRR estimates (Table 4). More precisely, for SCR = 0.0036, one would need to sample at least 1,000 individuals to ensure that chance is smaller than 10% whereas for SCR = 0.0108, the same is achieved for sample sizes of no less than 500 individuals. In practice, these problematic data sets imply that the corresponding SCR estimates underestimate the true SCR that generated the data (Table 4). This underestimation can be explained by the fact that just a few seronegative individuals may result from seroreversion events but they are wrongly assumed to have never been exposed to malaria parasites under a null SRR estimate. For higher transmission settings, the occurrence of these problematic data sets is minimal because the generated data has a good balance between the total number of seropositive and seronegative individuals.

**Table 4:** **Percentage of simulated data sets where SRR was estimated as 0 (%**_**ρ=0**_ **) and the bias of the corresponding SCR estimates taken as the percentage in relation to the true SCR**

**Population**	**Sample size**	**SCR = 0.0036**		**SCR = 0.0108**	
		**%**_**ρ=0**_	**Bias (%)**	**%**_**ρ=0**_	**Bias (%)**
Africa	250	28.6	−25.7	16.0	−26.1
	500	21.9	−25.3	8.4	−26.6
	1,000	13.1	−25.6	2.0	−25.6
	2,500	3.7	−25.2	0.1	−26.4
	5,000	0.6	−24.4	0.0	N/A
	10,000	<0.1	−22.4	0.0	N/A
Elsewhere	250	27.0	−29.6	14.6	−30.5
	500	20.1	−29.4	6.1	−30.2
	1,000	11.2	−29.2	1.5	−30.7
	2,500	3.0	−28.6	<0.1	−32.2
	5,000	0.4	−28.8	0.0	N/A
	10,000	0.0	N/A	0.0	N/A

Bias was defined as the difference between the mean of the corresponding estimates and the true value of SCR. The true SRR that generated the data sets was fixed at 0.017.

Approximated sample sizes were calculated using data simulation coupled with a regression model relating precision to sample size (Table 5); see Additional file 5 for the respective simulation results. Three key observations can be highlighted. Firstly, as found for known SRR, the same qualitative behavior between sample size and transmission intensity was found irrespective of the population under study. More precisely, the sample sizes increase from very low to moderate transmission and decrease from then on. Secondly, the necessity of estimating an additional parameter from the data brought more uncertainty over SCR estimation, thus, increasing the previous sample sizes for known SRR. In this case, the difference in sample sizes assuming or not a known SRR decreases with transmission intensity. On one extreme, for SCR = 0.0036, the sample sizes for relative precisions of 1, 0.75, 0.50 and 0.25 are now 2,193, 5,127 and >10,000, respectively, in comparison to 315, 549, 1163 and 4968 assuming a known SRR. On the other extreme, for SCR = 0.29, the sample sizes do not differ substantially assuming or not known SRR: 213, 267, 542, and 1,927 (unknown SRR) versus 151, 233, 461, and 1,670 (known SRR). Thirdly, for the same relative precision, African studies are most likely to require lesser individuals than their counterparts conducted elsewhere. This is in clear contrast to above results for known SRR where African studies would only have decreased sample sizes in high transmission intensities. The explanation for this result is unclear but it might be related again to the underlying age distribution. When SRR is unknown, the bulk of the information on SCR seems to come from young individuals and, if so, African populations have a higher proportion of individuals with that age. Finally, it is worth noting that, since the sample sizes were calculated using the same relative precision, the above-mentioned results for known SRR on comparing African to non-African studies are still valid for unknown SRR.

**Table 5:** **Approximate sample sizes for controlling precision of SCR estimates under of the assumption of unknown SRR where the true SRR was fixed at 0.017**

**Relative length**	**SCR**	**Sample size**	
		**Africa**	**Elsewhere**
1.00	0.0036	1,340	1,487
	0.0108	507	563
	0.0324	228	248
	0.0969	201	204
	0.2900	213	298
0.75	0.0036	2,193	2,414
	0.0108	871	980
	0.0324	323	379
	0.0969	240	285
	0.2900	267	474
0.50	0.0036	5,127	5,803
	0.0108	1,759	1,974
	0.0324	784	918
	0.0969	447	615
	0.2900	542	938
0.25	0.0036	>10,000	>10,000
	0.0108	7,910	8,461
	0.0324	2,676	3,077
	0.0969	1,746	2,077
	0.2900	1,927	3,057

## Discussion

In this paper, two sample size calculators for estimating antibody SCR were proposed. The first calculator is based on the assumption of known SCR and, because of that, it implies smaller sample sizes in relation to a situation where SCR is assumed to be unknown. Obtaining smaller sample size is important for studies where ethical issues, limited human and economic resources, or time constraints might be in place. However, this calculator requires fixing SRR at a given constant. In this regard, the current knowledge of SRR is still limited. Firstly, this parameter has only been measured indirectly by means of fitting the reverse catalytic models to data. Secondly, there might be age differences in seroreversion but seropositivity data appears to not have enough information for its detection [1]. Therefore, considering SRR at a fixed constant is a pragmatic choice not also for data analysis but also for sample size calculation. Notwithstanding this pragmatism, current estimates of SRR [1,7,13] are of the same of magnitude of the one used here and, therefore, the calculated sample sizes would appear to be reliable in general. However, for the matter of precision, sample size determination is recommended to be performed using a predefined SRR estimate from a reliable source. An obvious source of information can be data from another population but with similar malaria transmission intensity and host factors. Another possible source of information is to use existing data from past surveys taken from the same population, as reported in a recent study from Kenya [26]. Statistically speaking, a more coherent and elegant way to incorporate prior information in sample size determination is via Bayesian methodology as done elsewhere for estimating proportions (or prevalences) [27,28]. Although appealing, this approach would not appear to attract much attention of malaria epidemiologists, as suggested by the scarce number of studies applying such alternative approach to data analysis.

The basic idea underlying the first sample size calculator is to apply a back-transformation to the confidence interval for SP. The reliability of this method is then critically dependent not only on the statistical performance of the chosen SP confidence interval (in this case, the Wald Score corrected for continuity), but also on the degree of similarity between the age distribution used in the sample size determination and the one to be obtained upon sample collection. In terms of the Wald confidence interval using a continuity correction, it is one among more than twenty methods proposed to construct confidence interval for a proportion [23]. A recent study compared seven of these methods in terms of sample size determination for estimating a proportion [10]. General guidelines are not easy to put forward because they depend not only on the different criteria on how to deal with eventual problems of under or overcoverage of the corresponding confidence intervals, but also on the underlying proportion of the population under study. Notwithstanding this problem, these authors showed that, for a given absolute precision and a proportion between 0.01 and 0.90, the sample sizes from different methods do not deviate more than 40 sampling units. This result is expected to hold true for SCR estimation, but might require large-enough sample sizes where a linear approximation can be invoked between SCR and SP. With the respect to the age distributions used here, official statistics showed a clear distinction between African and non-African populations. However, these age distributions report to the respective overall populations and, thus, slight differences are expected to be seen between these whole-population-based distributions and the corresponding ones for the rural areas where malaria is more prevalent. Although a case-by-case approach is recommended, these differences are most likely to be related to a higher number of older individuals living in urban population that, in general, have better access to health care. Other factors related to sampling feasibility might also introduce some bias in the sampled age distribution, such as using schools surveys or collecting household-consented data that led to a slightly overrepresentation of school-aged children (5–18 years old) in recent studies [9,29,30]. Notwithstanding these putative differences between official and sampled age distributions, there is a good agreement between the age distributions used here and the ones found across a series of recent cross-sectional studies [31-33]. Thus, the calculated sample sizes would appear to be reliable for planning future surveys not using age stratification. A natural follow up of this work is then to perform sample size determination on alternative sampling strategies that may necessitate targeting or oversampling specific age groups. In theory, stratified sampling, if done intelligently, is known to improve precision of the ensuing estimates of the population prevalence [34]. Since the first sample size calculator is based on the confidence interval for SP, the sample sizes of age-adjusted sampling strategies should be decreased in relation to the ones calculated here. The optimal age stratification in terms of minimum sample size is one among other questions to be explored in a near future.

The second sample size calculator relates to the most general situation of a unknown SRR. Although general, this method only provides approximate sample sizes because it uses simulation coupled with a regression model predicting the expected precision as function of the sample size. As expected, the additional requirement of estimating SRR results in larger sample sizes in comparison to the ones derived from a known SRR. The simulation results highlighted the possibility of generating data sets from low transmission settings where one does not have enough information to estimate the SRR, thus, introducing significant negative biases on the SCR estimates. To minimize the occurrence of such situations, sample sizes of no less than 1,000 and 500 are recommended for EIR = 0.01 and 0.1, respectively. It is worth noting that there are many combinations of transmission intensities and relative precisions leading to sample sizes of more than 1,000 individuals. This relatively intensive sampling is particularly important for studying populations close to malaria elimination (SCR ≤ 0.0108). As a statistical advantage, a large sample size diminishes the chance of underestimating SCR due to null SRR estimates. However, large community-based surveys are usually seen as financially and logistically demanding enterprises and school or health centre surveys may be more pragmatic. As with a conventional metric like parasite rate, the relative advantages and disadvantages of a relatively small community-based survey and a large study using a more convenient sampling approach need to be properly balanced. Additionally the simulation algorithm for calculating precision assumes a population of infinite size. This assumption is reasonable in highly dense populations living in small areas where malaria transmission is expected to be more homogeneous. However, this is uncommon with heterogeneity in population density and malaria transmission more likely to be the norm especially at low transmission. The corresponding sample size will need to be inflated if one is to unravel subpopulations with subtle differences in malaria exposure, as observed in different studies [1,7,13]. Finally, a large sample size might not be feasible in intrinsically small populations, such as the ones living in islands [4,9]. In that case, the precision is in fact increased in relation to the one calculated from infinite-size population and, thus, the proposed sample size calculator would lead to oversampling. However, if there are no dramatic cost restrictions, oversampling might overcome eventual losses of precision due to the occurrence of missing data.

It is also important highlighting the fact that the SCR and SRR used here are for the merozoite surface protein-1 (MSP1) antigen. Another well-characterized antigen is the *P. falciparum* apical membrane antigen-1 (AMA1). Current SCR and SRR estimates are different for these two antigens due to their inherent immunogenicity and half-life exposed to the immune system [8] with a higher SCR for AMA1 compared to its MSP1 counterpart. As a direct consequence of this observation, smaller sample sizes will be required for AMA1-based studies. There is relatively little data for other antigens though variation in seroconversion rates has been reported [35,36]. Practically to overcome issues around antigenic variation and differential population reactivity (e.g., due to genetics), a combination of antigens are used and sample sizes would be derived from the most immunogenic component.

In conclusion, this paper described relatively straightforward approaches to calculating the sample size for estimating SCR. The methods assume data derived from areas with stable transmission, standard population age distributions and community-based surveys with no age stratification. Several caveats relating to survey design, antibody reversion rates and antigen choice were presented to allow an appreciation of the complexity of the issue. Pragmatically however, the results suggest that SCR estimation can be readily incorporated into the design of most malariometric studies and this will be of particular use in populations with low malaria endemicity. Further work is needed to assess the sample size requirements for estimating any change in transmission with serology.

## Additional files

Additional file 1:
**Relationship between altitude and different malariometrics in northeast Tanzania: altitude versus EIR (A), altitude versus SCR (B), altitude versus PR**_**04**_ **(C).**
Additional file 2:
**Age distributions of different countries from West Africa, East Africa, South America and Southeast Asia.**
Additional file 3:
**Midpoints of confidence intervals for SCR as function of the sample size (A) and the derivative function of SP in relation to SCR (B).**
Additional file 4:
**Absolute SCR, EIR and SP ranges using the sample sizes shown in Table** 3 **and 99% confidence level for the respective intervals.**
Additional file 5:
**Results of the simulation study when SRR is unknown.** The true SRR of the population was setup at 0.017.

## Abbreviations

EIR: entomological inoculation rate
PR: parasite rate
SP: seroprevalence
SCR: seroconversion rate (*λ*)
SRR: seroreversion rate (*ρ*)
