# Supplementary figures and images for: Sample size determination for estimating antibody seroconversion rate under stable malaria transmission intensity
Source: Malar J. 2015 Apr 3;14:141. doi: 10.1186/s12936-015-0661-z (PMC4419413; doi:10.1186/s12936-015-0661-z)

**A**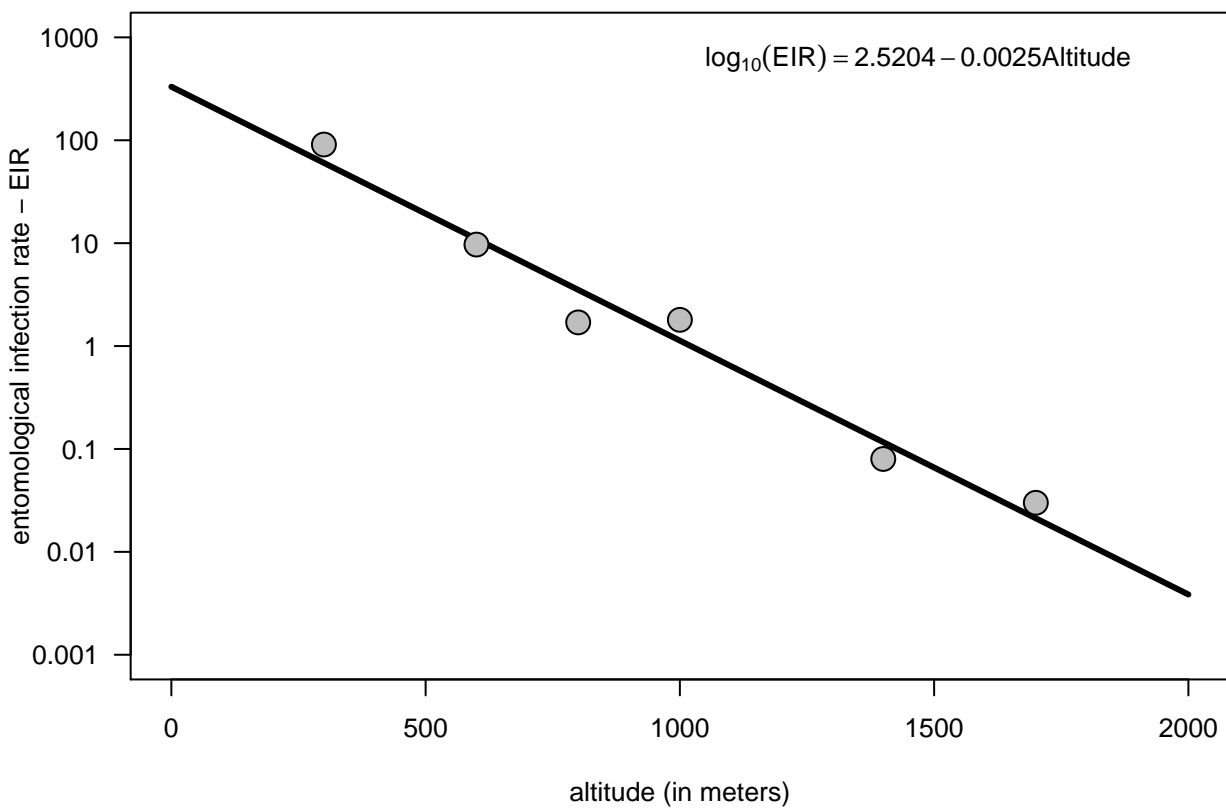**B**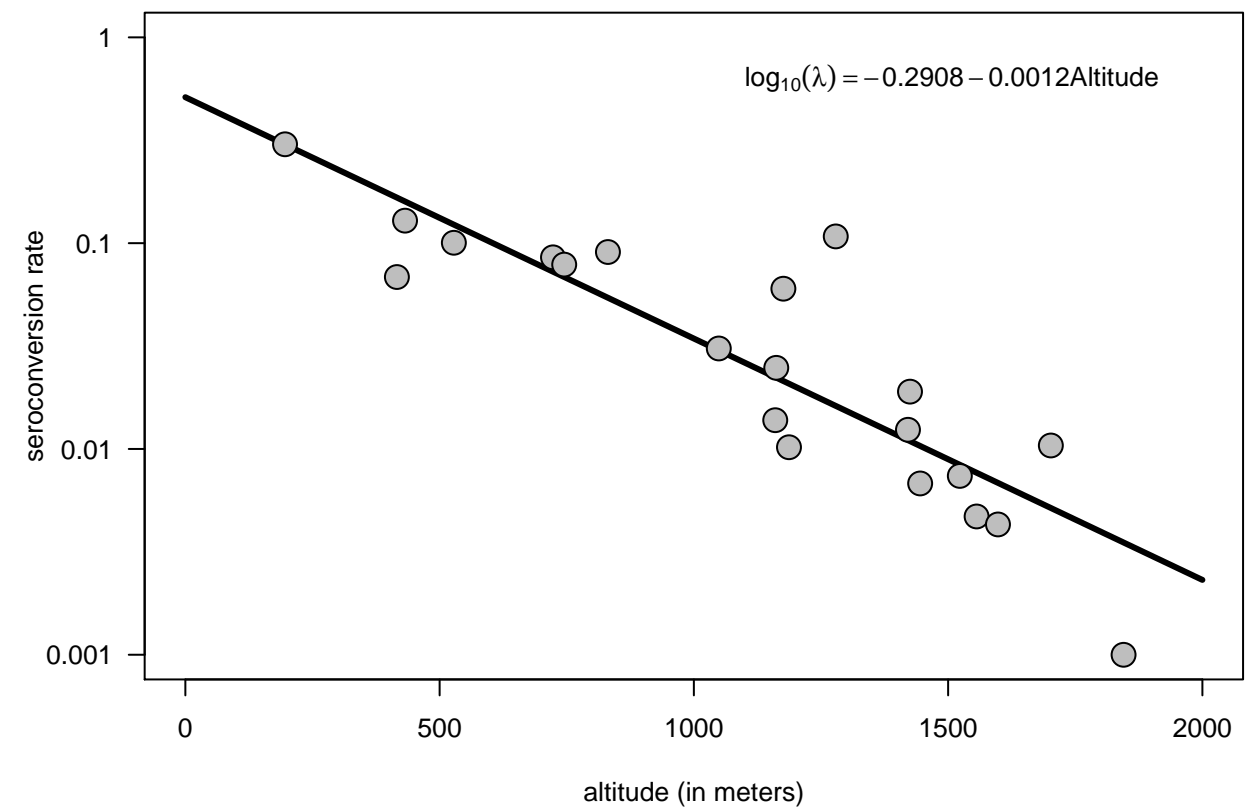**C**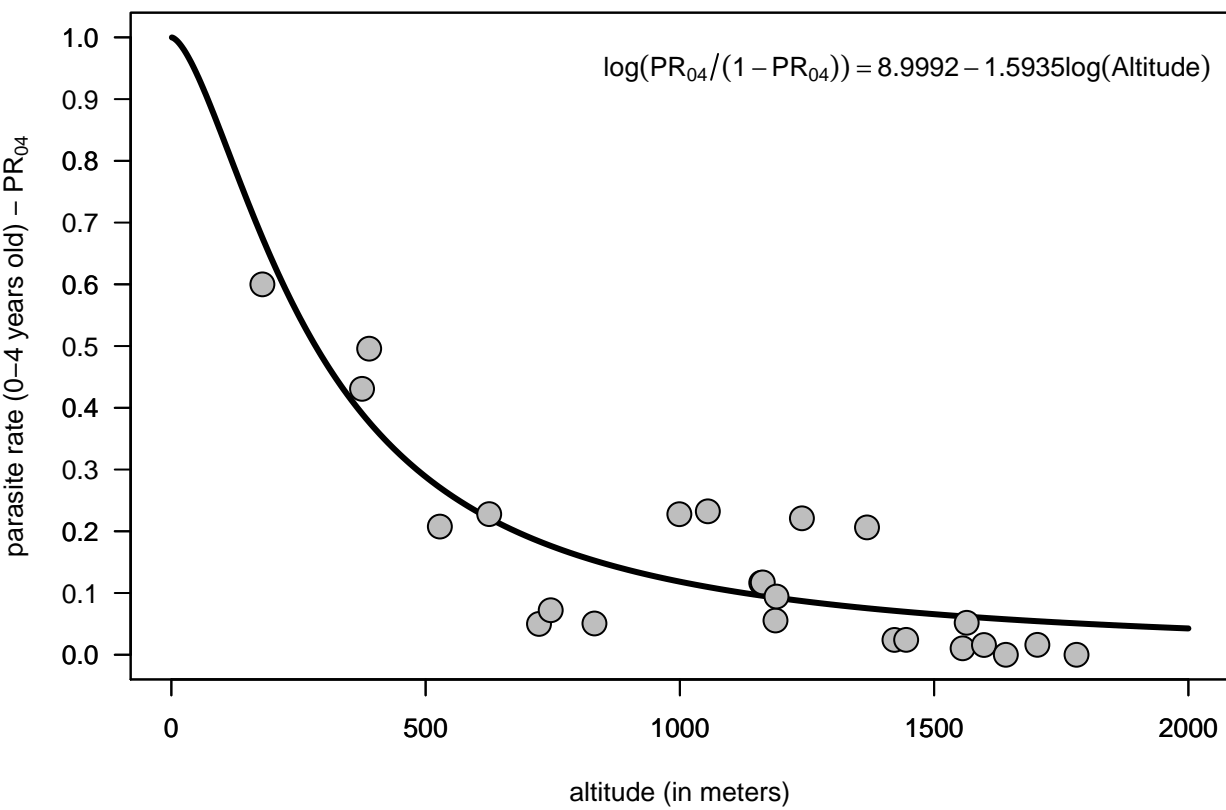

Supplement: Additional file 1: — Relationship between altitude and different malariometrics in northeast Tanzania: altitude versus EIR (A), altitude versus SCR (B), altitude versus PR 04 (C). [file 12936_2015_661_MOESM1_ESM.pdf]

**West Africa**

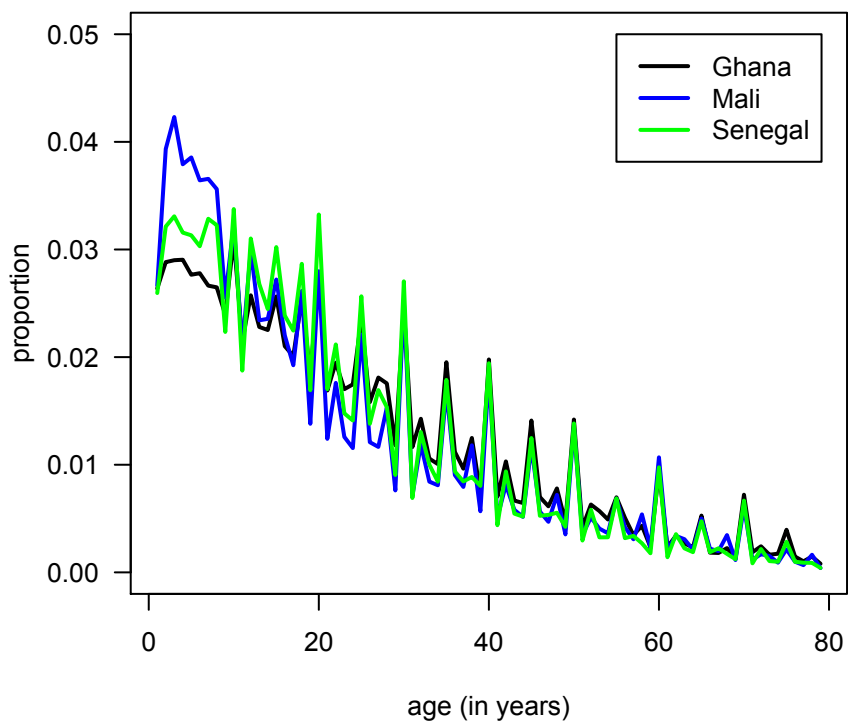

**East Africa**

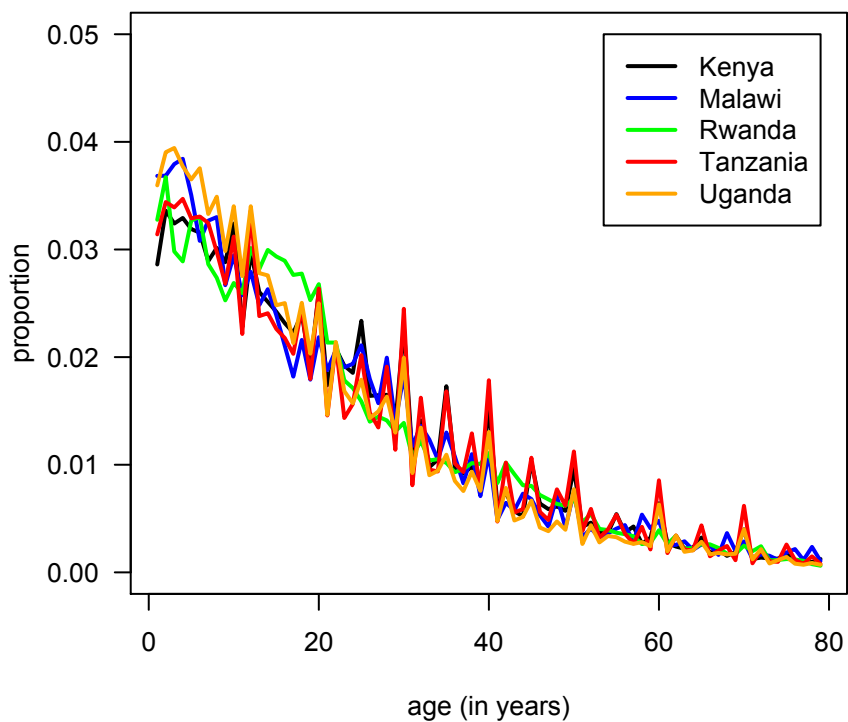

**Southeast Asia**

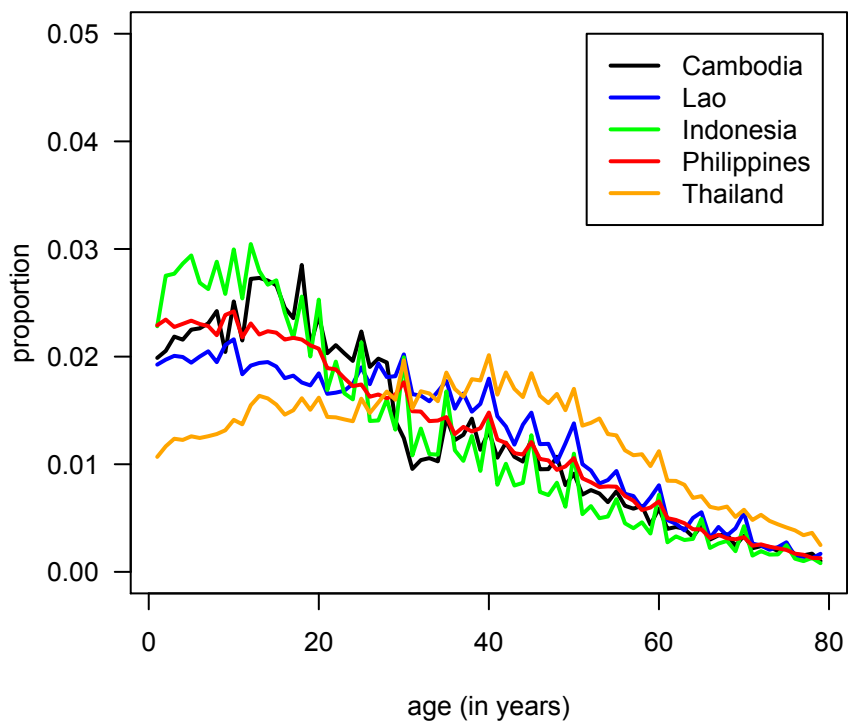

**South America**

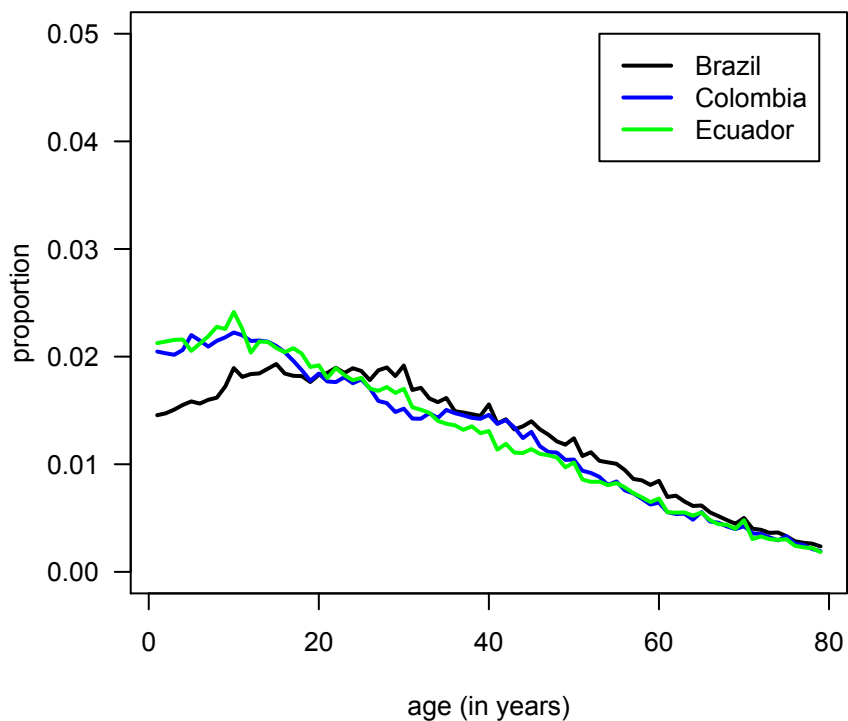

Supplement: Additional file 2: — Age distributions of different countries from West Africa, East Africa, South America and Southeast Asia. [file 12936_2015_661_MOESM2_ESM.pdf]

**A****Africa**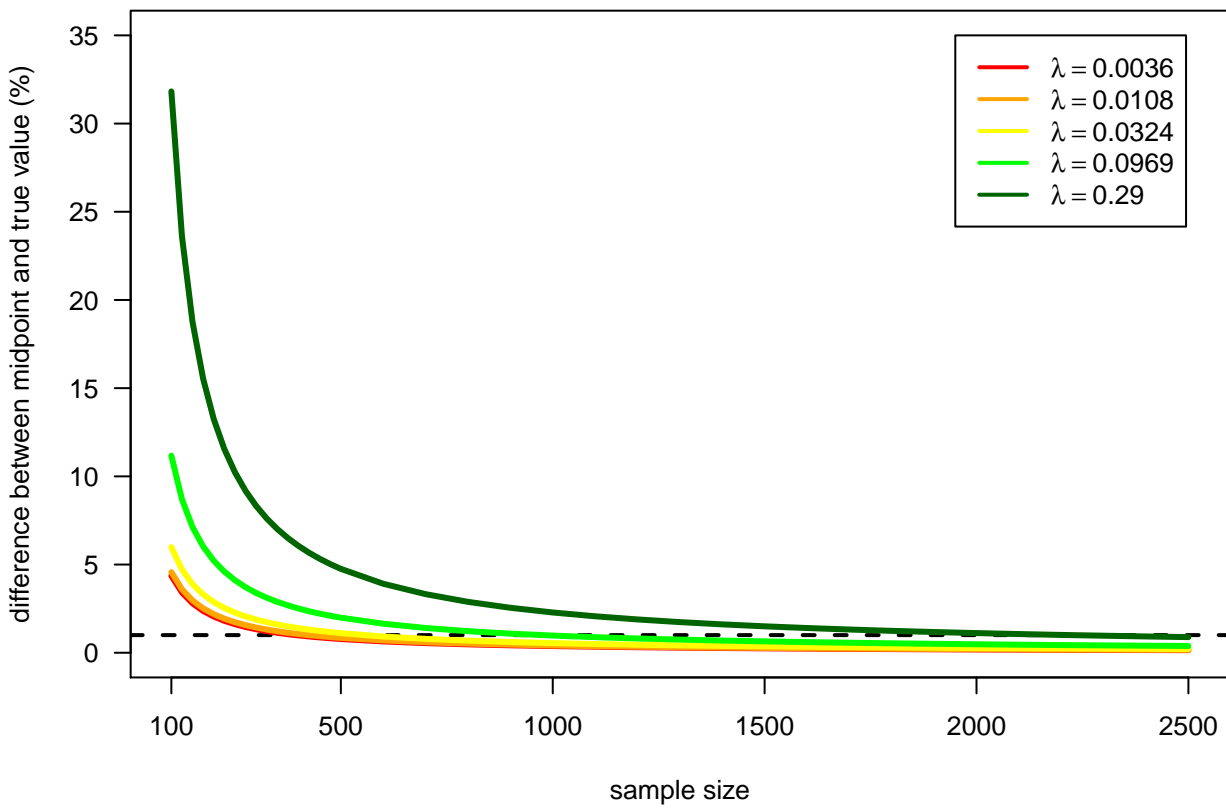**Southeast Asia & South America**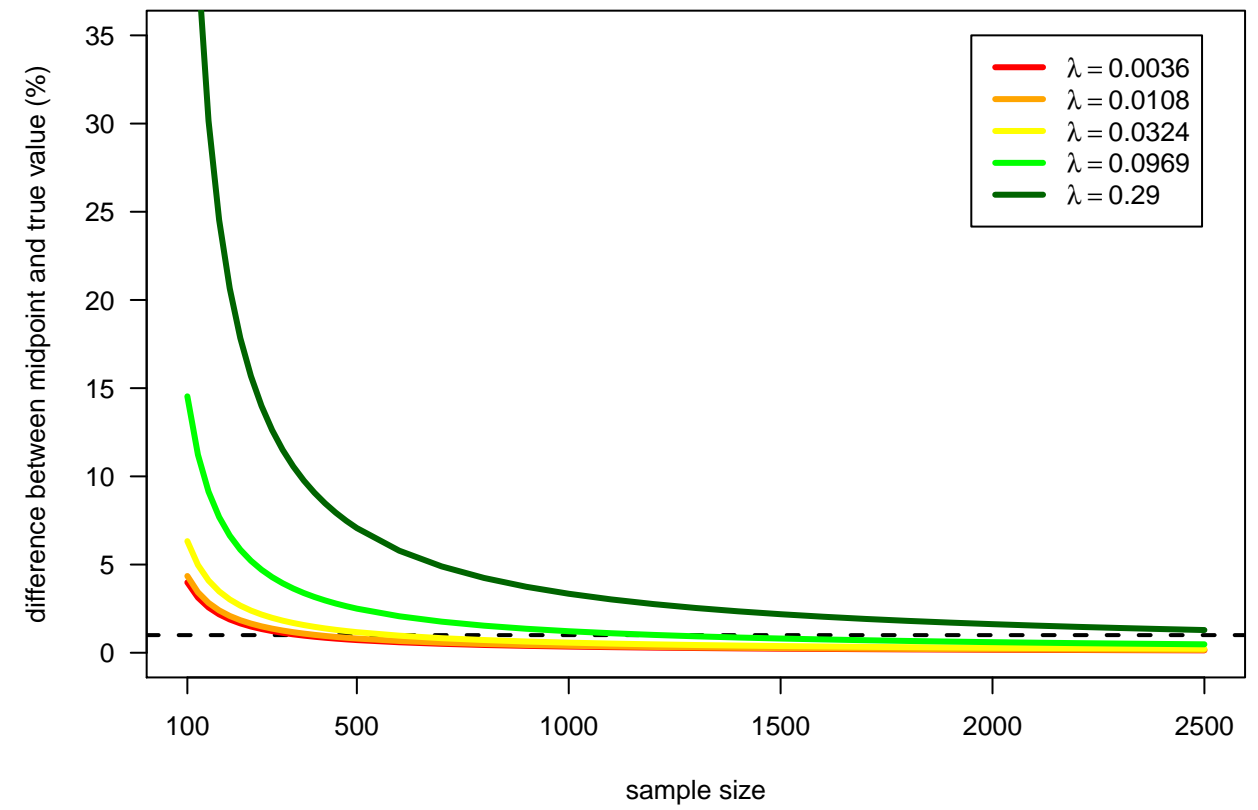**B**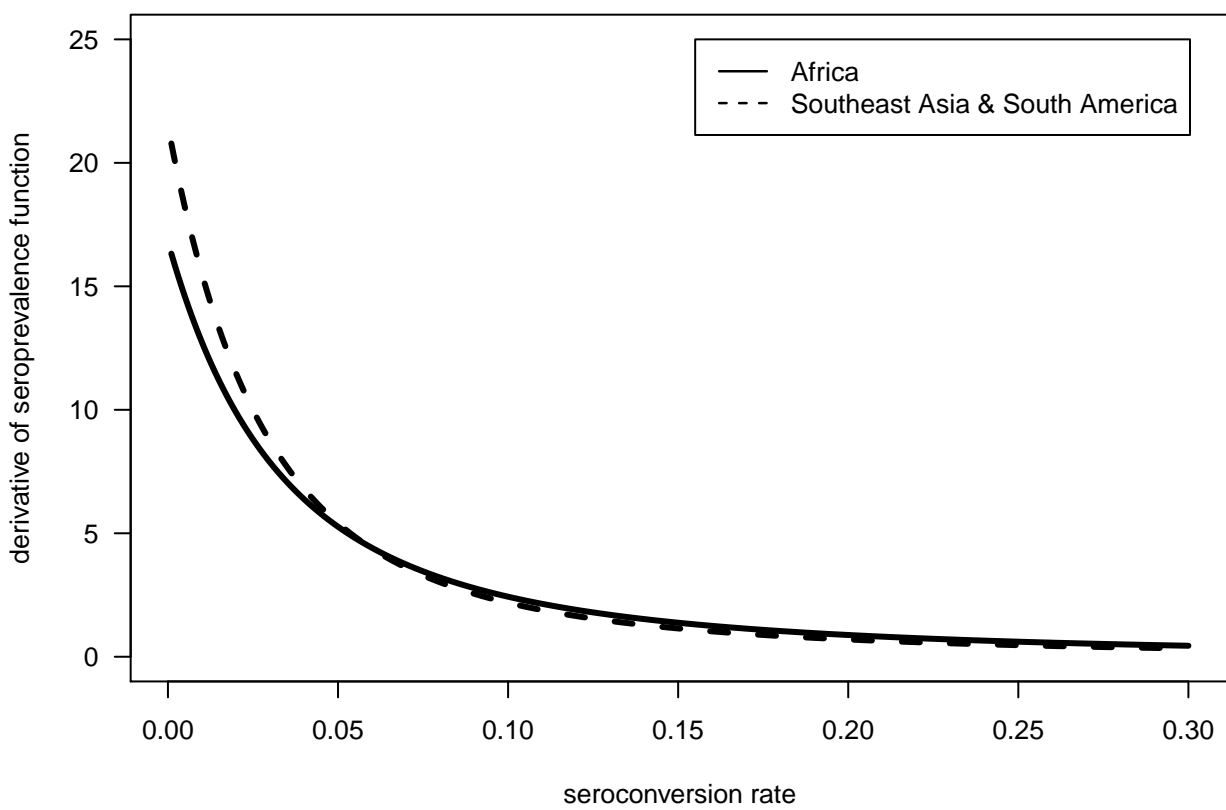

Supplement: Additional file 3: — Midpoints of confidence intervals for SCR as function of the sample size (A) and the derivative function of SP in relation to SCR (B). [file 12936_2015_661_MOESM3_ESM.pdf]
