# Supplementary material for: Sample size determination for estimating antibody seroconversion rate under stable malaria transmission intensity
Source: Malar J. 2015 Apr 3;14:141. doi: 10.1186/s12936-015-0661-z (PMC4419413; doi:10.1186/s12936-015-0661-z)
Supplement: Additional file 4: — Absolute SCR, EIR and SP ranges using the sample sizes shown in Table 3 and 99% confidence level for the respective intervals. [file 12936_2015_661_MOESM4_ESM.pdf]

Additional file 4

Absolute seroconversion rate (SCR), entomological inoculation rate (EIR) and seroprevalence ranges using the sample sizes shown in Table 3 and 99% confidence level for the respective intervals.

population	SCR	sample size	absolute SCR range	absolute EIR range	seroprevalence range
Africa	0.0036	281	0.0014–0.0062	0.00–0.03	0.023–0.095
		494	0.0019–0.0055	0.00–0.03	0.031–0.085
		1107	0.0025–0.0049	0.00–0.02	0.040–0.076
		4602	0.0030–0.0042	0.01–0.01	0.049–0.066
	0.0108	116	0.0047–0.0189	0.02–0.34	0.074–0.248
		203	0.0060–0.0167	0.03–0.27	0.093–0.225
		451	0.0075–0.0146	0.05–0.20	0.113–0.202
		1780	0.0091–0.0126	0.07–0.15	0.135–0.179
	0.0324	68	0.0165–0.0599	0.26–3.79	0.222–0.523
		115	0.0195–0.0519	0.37–2.81	0.254–0.486
		251	0.0232–0.0446	0.53–2.05	0.289–0.446
		979	0.0274–0.0381	0.75–1.48	0.326–0.406
	0.0969	75	0.0573–0.1940	3.46–43.92	0.512–0.796
		121	0.0636–0.1631	4.30–30.58	0.540–0.764
		251	0.0719–0.1368	5.55–21.20	0.572–0.727
		949	0.0828–0.1148	7.45–14.72	0.608–0.688
	0.2900	145	0.1863–0.6188	40.35–492.04	0.789–0.938
		223	0.2005–0.5084	47.02–326.74	0.802–0.922
		443	0.2206–0.4179	57.36–217.23	0.819–0.904
		1625	0.2493–0.3456	74.05–146.18	0.838–0.883
SEA+SA	0.0036	223	0.0014–0.0062	0.00–0.03	0.030–0.120
		402	0.0020–0.0055	0.00–0.03	0.041–0.108
		861	0.0024–0.0048	0.00–0.02	0.051–0.096
		3570	0.0030–0.0042	0.01–0.01	0.062–0.084
	0.0108	94	0.0048–0.0191	0.02–0.35	0.095–0.306
		165	0.0061–0.0168	0.03–0.27	0.119–0.278
		367	0.0075–0.0146	0.05–0.20	0.143–0.250
		1481	0.0091–0.0126	0.07–0.15	0.169–0.222
	0.0324	62	0.0172–0.0610	0.28–3.94	0.283–0.608
		104	0.0200–0.0524	0.39–2.88	0.316–0.567
		222	0.0234–0.0449	0.53–2.08	0.353–0.525
		864	0.0275–0.0382	0.75–1.48	0.394–0.481
	0.0969	86	0.0596–0.1996	3.75–46.57	0.601–0.851
		136	0.0652–0.1659	4.52–31.71	0.625–0.823
		276	0.0727–0.1381	5.68–21.61	0.653–0.792
		1031	0.0830–0.1151	7.49–14.80	0.685–0.757
	0.2900	202	0.1893–0.6275	41.74–506.58	0.844–0.954
		308	0.2027–0.5130	48.09–332.93	0.853–0.943
		606	0.2218–0.4199	58.04–219.33	0.865–0.929
		2203	0.2497–0.3461	74.26–146.61	0.880–0.913
