# Supplementary material for: Sample size determination for estimating antibody seroconversion rate under stable malaria transmission intensity
Source: Malar J. 2015 Apr 3;14:141. doi: 10.1186/s12936-015-0661-z (PMC4419413; doi:10.1186/s12936-015-0661-z)
Supplement: Additional file 5: — Results of the simulation study when SRR is unknown. The true SRR of the population was setup at 0.017. [file 12936_2015_661_MOESM5_ESM.pdf]

Additional file 5

Results of the simulation study when seroreversion rate is assumed to be unknown. The true seroreversion rate was fixed at 0.017.

population	SCR	sample size	relative length	absolute SCR range	EIR range	seroprevalence range
Africa	0.0036	250	4.38	0.0016–0.0174	0.00–0.29	0.027–0.232
		500	1.95	0.0020–0.0090	0.00–0.07	0.033–0.134
		1000	1.18	0.0023–0.0066	0.00–0.04	0.037–0.100
		2500	0.72	0.0026–0.0052	0.01–0.02	0.042–0.081
		5000	0.53	0.0028–0.0047	0.01–0.02	0.045–0.074
		10000	0.37	0.0030–0.0043	0.01–0.02	0.048–0.068
	0.0108	250	1.52	0.0064–0.0228	0.04–0.51	0.098–0.286
		500	1.01	0.0070–0.0179	0.04–0.31	0.107–0.237
		1000	0.68	0.0079–0.0152	0.06–0.22	0.118–0.208
		2500	0.43	0.0087–0.0134	0.07–0.17	0.130–0.188
		5000	0.30	0.0093–0.0126	0.08–0.15	0.137–0.178
		10000	0.22	0.0097–0.0120	0.09–0.13	0.142–0.171
	0.0324	250	0.90	0.0219–0.0509	0.47–2.7	0.277–0.481
		500	0.62	0.0243–0.0444	0.58–2.04	0.299–0.445
		1000	0.43	0.0263–0.0403	0.68–1.66	0.316–0.420
		2500	0.27	0.0284–0.0371	0.80–1.40	0.334–0.399
		5000	0.19	0.0295–0.0356	0.87–1.29	0.343–0.389
		10000	0.13	0.0304–0.0346	0.92–1.21	0.350–0.382
	0.0969	250	0.71	0.0710–0.1400	5.41–22.24	0.568–0.732
		500	0.48	0.0777–0.1244	6.53–17.39	0.592–0.706
		1000	0.34	0.0824–0.1150	7.37–14.77	0.607–0.688
		2500	0.21	0.0878–0.1077	8.41–12.89	0.623–0.673
		5000	0.14	0.0903–0.1043	8.93–12.06	0.630–0.665
		10000	0.10	0.0921–0.1022	9.31–11.55	0.635–0.660
	0.2900	250	0.80	0.2073–0.4394	50.39–241.12	0.808–0.909
		500	0.52	0.2288–0.3804	61.91–178.55	0.825–0.894
		1000	0.36	0.2454–0.3500	71.62–150.14	0.836–0.884
		2500	0.22	0.2605–0.3250	81.16–128.65	0.845–0.875
		5000	0.16	0.2689–0.3145	86.65–120.09	0.849–0.871
		10000	0.11	0.2747–0.3072	90.62–114.40	0.852–0.868
SEA+SA	0.0036	250	5.29	0.0017–0.0208	0.00–0.42	0.028–0.266
		500	2.24	0.0020–0.0100	0.00–0.09	0.033–0.147
		1000	1.27	0.0022–0.0068	0.00–0.04	0.036–0.104
		2500	0.78	0.0025–0.0053	0.01–0.02	0.041–0.083
		5000	0.54	0.0028–0.0047	0.01–0.02	0.045–0.074
		10000	0.39	0.0030–0.0044	0.01–0.02	0.048–0.069
	0.0108	250	1.64	0.0063–0.0241	0.03–0.57	0.097–0.297
		500	1.06	0.0070–0.0184	0.04–0.32	0.106–0.242
		1000	0.73	0.0076–0.0155	0.05–0.23	0.115–0.211
		2500	0.45	0.0086–0.0135	0.07–0.17	0.128–0.189
		5000	0.32	0.0093–0.0127	0.08–0.15	0.137–0.179
		10000	0.22	0.0097–0.0121	0.08–0.14	0.142–0.172
	0.0324	250	0.99	0.0213–0.0534	0.44–2.99	0.271–0.493
		500	0.67	0.0236–0.0454	0.55–2.13	0.293–0.450
		1000	0.47	0.0259–0.0410	0.66–1.73	0.313–0.424
		2500	0.29	0.0281–0.0374	0.79–1.43	0.332–0.401
		5000	0.20	0.0294–0.0359	0.86–1.31	0.342–0.391
		10000	0.14	0.0302–0.0349	0.91–1.23	0.349–0.383
	0.0969	250	0.82	0.0681–0.1480	4.96–24.97	0.558–0.744
		500	0.56	0.0755–0.1295	6.15–18.90	0.584–0.715
		1000	0.37	0.0809–0.1172	7.10–15.37	0.602–0.693
		2500	0.24	0.0862–0.1095	8.11–13.33	0.618–0.677
		5000	0.17	0.0892–0.1057	8.70–12.39	0.627–0.669
		10000	0.12	0.0914–0.1030	9.16–11.73	0.633–0.662
	0.2900	250	1.15	0.1944–0.5272	44.11–352.37	0.797–0.925
		500	0.73	0.2152–0.4255	54.47–225.48	0.814–0.905
		1000	0.47	0.2355–0.3708	65.73–169.31	0.829–0.891
		2500	0.29	0.2523–0.3374	75.91–139.07	0.840–0.880
		5000	0.21	0.2629–0.3228	82.69–126.84	0.846–0.874
		10000	0.14	0.2703–0.3121	87.59–118.22	0.850–0.870
